# Professionals’ Self-Reported Difficulties towards Integrating Dual Task Training in Care for People with Parkinson’s Disease

**DOI:** 10.3390/ijerph19031281

**Published:** 2022-01-24

**Authors:** Josefa Domingos, John Dean, Júlio Belo Fernandes, Catarina Godinho

**Affiliations:** 1 Grupo de Patologia Médica, Nutrição e Exercício Clínico (PaMNEC) do Centro de Investigação Interdisciplinar Egas Moniz (CiiEM), 2829-511 Almada, Portugal; jfernandes@egasmoniz.edu.pt (J.B.F.); cgodinho@egasmoniz.edu.pt (C.G.); 2 Department of Neurology, Radboud University Medical Center, Donders Institute for Brain, Cognition and Behaviour, 6500 HB Nijmegen, The Netherlands; 3 Triad Health AI, Aurora, CO 80012, USA; john@johnmdean.com

**Keywords:** Parkinson’s disease, exercise, dual task training, difficulties, health professionals

## Abstract

Background: Despite the growing use of dual task training (DTT) in clinical practice with people with Parkinson Disease (PD), there is still limited evidence on how to best implement it. Data regarding professionals’ difficulties when integrating such practices are critical as a first step to generate further guidance on how to apply it. The aim of this study was to identify the difficulties perceived by professionals to integrate dual task in their practice. Methods: A descriptive, observational and cross-sectional study was conducted using a web-based survey. Convenience sampling was used to recruit exercise and healthcare professionals working with people with PD through various social media channels. Data were collected and then analyzed thematically using the method of constant comparisons. The study report follows the consolidated criteria for reporting qualitative research (COREQ) checklist. Results: Of the 205 eligible responses, 68.8% were Physiotherapist. The majority of the participants reported having Parkinson-specific training (91.7%) and 59.0% applied DTT in individual one-on-one sessions. We identified ten categories of difficulties faced by professionals. Conclusions: Professionals struggle to integrate DTT into PD clinical care. Challenges were identified and the most significant refer to difficulties in managing the chronicity of the disease and lack of patient compliance with home exercises. Understanding current challenges towards dual task exercise will help to reflect upon strategies to be applied effectively and safe.

## 1. Introduction

Parkinson’s disease (PD) is one of the fastest-growing neurological diseases [1] affecting an estimated 10 million people [2,3]. PD is a complex disease affecting posture, gait, and speech with far reaching impact on daily living activities [4,5,6]. In addition to these motor disturbances, people with PD may experience a wide range of non-motor symptoms, such as gastrointestinal disturbances, autonomic dysfunctions, sleep problems, sensory manifestations, neuropsychiatric and cognitive symptoms negatively contributing to severe disability and reduced life expectancy [7].

Impairments like gait, balance and cognition are known to be exacerbated under dual-task conditions, which occur when individuals attempt to simultaneously perform cognitive and motor tasks [8,9,10]. For example, in gait impairments, people with PD performing a concurrent task while walking experience more freezing episodes and a reduction in step length and gait velocity, when compared to only walking [8,11].

Dual task training (DTT) is a promising new intervention method that has been shown to improve such difficulties in elderly adults [12,13,14] and in PD [15,16,17,18,19]. Research supports that using dual task during training in PD is safe and has benefits related to gait (gait speed, step length and cadence) and balance (mediolateral and anteroposterior balance in closed-eyes tests) [20]. It is rapidly gaining an important role as part of the care for people PD, particularly in the treatment of gait disturbance, impaired balance and risk of falls [20,21].

With the proliferation of such practice and an increase in the diversity of providers delivering such care, recognizing the difficulties perceived by professionals will help identify solutions to provide the best quality care when integrating DTT in PD. Here we aim to explore the difficulties faced by healthcare and exercise professionals when providing DTT with people with PD. We offer an international landscape of dual task training for people with PD, that may guide potential reflections on solutions for the difficulties identified to integrate DTT in care.

## 2. Materials and Methods

### 2.1. Study Design

This descriptive, observational and cross-sectional study was conducted using a web-based survey. The study report follows the consolidated criteria for reporting qualitative research (COREQ) checklist.

### 2.2. Sampling and Recruitment

The study population consists of healthcare and exercise professional’s that integrate some form of dual task into their practice with people with PD. Convenience sampling was used to recruit participants via various social media channels (via Facebook professional groups, WhatsApp, and Instagram). The invitation contained information regarding the study and a link to access the survey. We included participants if they were a healthcare or exercise professional, had previous experience working with people with PD, and integrate some form of DTT into their practice with people with PD. Participants were excluded if they were unwilling to participate in the study and to provide informed consent to participate in the study.

### 2.3. Data Collection

Data were collected from 1 May 2021, until 30 August 2021. The survey was hosted on Google^TM^ Forms website. It consisted of two pages. The study information page and a second page contained questions regarding the participants sociodemographic status, primary clinical setting and years of practice and explored the difficulties faced by healthcare and exercise professionals when providing DTT to people with PD. The total survey length was approximately 8 min.

The survey pre-testing was made with ten participants. Participants were questioned to know their perceptions about the survey. The instrument was considered to be sufficiently clear, objective, comprehensive, and did not present questions that could be ambiguous or equivocal. This feedback allowed researchers to conclude that the survey proved to be suitable for this study.

### 2.4. Data Analysis

Data were extracted to a spreadsheet. Using the IBM Statistic Package for the Social Sciences software, (IBM Corp. Released 2020. IBM SPSS Statistics for Windows, Version 27.0. Armonk, NY, USA: IBM Corp.) descriptive statistics were performed to analysed quantitative data. Only complete questionnaires were analysed.

Textual data from open-ended question was exported to QDA Miner Lite database and analysed using the process of content analysis as described by Braun, Clarke, Hayfield and Terry (2019) [22], which involved pre-analysis, encoding, categorisation, and interpretation of data. This process allowed us to identify categories that would answer the research question. The records were reviewed; the text was divided into meaning units of words, phrases and passages on the same topic. Using the participants’ own responses, codes were assigned to the meaning units and categories were identified reflecting differences and similarities in these responses. The different stages of analysis were separately done by two investigators and any differences were resolved by discussion.

### 2.5. Ethical Considerations

This study follows the principles of the Declaration of Helsinki. The Egas Moniz Research Ethics Board approved the ethical components of this study (Date: April 2021 ID: 964). On the first page of the survey, it was stated that participation was entirely voluntary. Participants were also free to, not reply to some questions, change, or review their responses, or voluntarily quit at any time. Informed consent was obtained before proceeding to the next page when participants answered “YES” to the form’s first question and agreed to participate in the study. Participants who answered “NO” to the informed consent question were directed to the end of the survey. Data was conducted in compliance with ethical principles guaranteeing the participants’ anonymity and confidentiality. Therefore, no personally-identifying information was collected, and no individual answers to the questionnaire will be accessible.

## 3. Results

### 3.1. Characteristics of the Participants

There were 205 eligible responses (Table 1), the majority from America (83.9%, of which 80.5% were from the USA, 2.9% from Canada, and 0.5% from Brazil), 68.8% were physiotherapists, followed by 11.7% occupational therapist and 6.3% speech therapist and 13.2% other exercise and clinical professionals.

The most common level of education was a Master’s degree (36.1%), followed by Doctoral degree (35.6%) and Postsecondary/Undergraduate (27.8%). Regarding professional experience, the total mean years of practice was 19.2 ± 11.1 (range 1–45 years). The mean years of practice specifically with people with PD was 12.5 ± 8.8 (range 1–40 years). The majority of the participants previously attended Parkinson specific training (91.7%) and employ DTT in individual one on one sessions (59.0%) while only 3.4% of the participants used it exclusively in group.

### 3.2. Key Themes from the Data

Data analysis revealed 4 different themes with ten categories of difficulties faced by healthcare and exercise professionals when providing DTT with people with PD (Table 2).

### 3.3. Manage the Disease Symptoms and Medication

#### 3.3.1. PD Signs and Symptoms

Participants revealed that dealing with the diverse signs and symptoms of the disease, particularly cognitive impairments and freezing of gait (FOG), can be challenging when providing DTT. Cognitive decline may limit the person’s capacity to divide (simultaneously) or to allocate appropriately resources when performing dual tasks. Freezing can be a severe symptom of PD that has been associated with worsening under dual task contexts. Visual and auditory cueing is often the treatment choice.

*“Cognitive impairment in people with Parkinson’s disease that compromise learning movements can be very challenging to deal with.”*

(S72)

*“It is difficult to deal with executive dysfunction; visual spatial difficulties; freezing of gait and with cognitive difficulties.”*

(S97)

#### 3.3.2. Medication Fluctuations

Participants reported difficulties managing the impact of medication effects and fluctuations. They also reported difficulties in coordinating training with medication on off times. Medication is considered effective in managing several PD symptoms, but many patients experience variations in their symptoms as the dosage of medication wears off. Motor or non-motor fluctuations can occur and be predictable (associated to time of intake of medication) but as disease progresses, they can become more and more unpredictable and occur suddenly, impacting their ability to perform the training programs.

*“If the person with Parkinson is off or on during their treatment session interferes with treatment, as well as some medication make clients nauseous or fatigued.”*

(S160)

*“Medication On Off times is difficult and coordinating exercise times with that.”*

(S174)

### 3.4. Patient Dynamics

#### 3.4.1. Poor Compliance

Participants stated that people with PD demonstrate poor levels of compliance with DTT, especially to follow through the exercise programs and remain compliant with home exercises. This is considered crucial in any rehabilitation process for carryover and transfer of learnings to real life situations.

*“Getting clients to understand the importance of exercising at home is very time consuming.”*

(S67)

#### 3.4.2. Motivation

Motivating people with PD to continue participate in regular exercise was a difficulty reported by participants. Despite evidence for the benefits of exercise in PD, patients have low levels of motivation and remain particularly sedentary.

*“Their own motivation to continue. Convincing them to work hard enough can be challenging.”*

(S96)

### 3.5. Training Dynamics

#### 3.5.1. Tailored Interventions

Adjusting exercises to each person’s physical and cognitive abilities, especially in group sessions, can be challenging for participants and thus sometimes they are unable to provide tailored interventions for each person.

*“The complexity of DTT sessions requires a lot of individual tailoring of program. Every Parkinson’s patient is different, we can’t generalize, more so in group settings.”*

(S102)

#### 3.5.2. Patient Safety

Participants reported difficulties in maintaining patient safety when using DTT because they feel that there is an increased risk of falls, especially during group sessions, where there is less supervision due to the lower ratio between client and healthcare and exercise professional.

*“Safety in group settings can be an issue. We need to be more on high alert due to increased falls risk in this cohort.”*

(S12)

#### 3.5.3. Keeping it Challenging and Engaging

Participants report struggling in inventing continuously new exercises (or adapting older ones) so that activities are challenging and engaging for people with PD. This is a relevant factor that can influence patient’s motivation and compliance to perform ongoing DTT exercise.

*“It’s difficult keeping it challenging and engaging. You need to create fun and meaningful tasks. Otherwise, they will not comply.”*

(S24)

### 3.6. External Factors

#### 3.6.1. Insurance Coverage

Insurance coverage was mentioned as a barrier to have adequate number of training sessions and time for each patient. Insurers can differ on how much they will pay for physical therapy, how long a policyholder can have it, and not cover all treatment plans.

*“Insurance limitations for desired intensity compliance. The limited number of visits we are allowed.”*

(S82)

#### 3.6.2. Environment

Participants reported environments not conducive to applying DTT in a proper and safe manner, often because the physical facilities do not lend themselves well to these type of interventions as there are frequently too many stimuli present in busy clinics and gyms.

*“I’ve also found that busy clinics tend to make it hard for people with PD to focus on what they’re doing.”*

(S112)

#### 3.6.3. Lack of Family Support

Respondents identified the lack of family support for follow through with home programs between therapy sessions as a key difficulty faced by healthcare and exercise professionals when providing DTT with people with PD.

*“The family support system is very important for a patient’s recovery. Getting the spouses/caregivers to help with home programs sometimes is challenging.”*

(S198)

## 4. Discussion

The use of DDT in people with PD is an emerging area of clinical interest but implementation is still a struggle [20,21,23]. This qualitative study has identified several difficulties reported by health and exercise professionals in applying DTT which are likely to influence the way care is applied by professionals in clinical practice. The most frequent difficulties reported by participants included issues with specific PD signs and symptoms (e.g., cognition and freezing), medication fluctuations and poor patient compliance and motivation. Respondents also reported difficulties with tailoring interventions (specifically for groups), keeping activities challenging and engaging, patient safety, insurance limitations, insufficient training environments, and lack of family support as other key barriers. Understanding the difficulties perceived by professionals when applying DTT will help guide integration of DTT exercise as an added tool within formal care programs commonly applied in PD. This study is the first to gather information from different countries regarding difficulties professionals have when applying DTT.

 

*Managing specific PD signs and symptoms*

PD is a complex disease and people with PD experience increasing disability as disease progresses making management more and more arduous [3,24]. Symptoms like cognitive decline and freezing of gait are particularly difficult to manage [25,26,27]. DTT will be more challenging to apply to people with profiles where learning may be sufficiently impaired to prevent or significantly limit an individual’s ability to learn strategies to divide attention in a safe and effective manner [28]. Research has shown that people with freezing of gait had smaller retention of the effects of the training program when compared to the patients without freezing [16,28,29]. Based on this observation, recommendations were provided, stating that patients with minimal or mild form of freezing of gait (occurring rarely and with short duration) with no cognitive impairment should start immediately with DTT. If patients have a moderate to severe freezing of gait (frequently occurring, long duration), without any cognitive problems, professionals should start training both tasks separately, and progress to training them simultaneously, if possible. Similarly, people with low cognitive functioning also benefited less from training tasks simultaneously and recommendations were to start with training both tasks apart and very slow progression to dual task [16,28,29].

Deficits in cognition may impact the individual’s ability to benefit from DTT. They may benefit more from developing compensatory strategies than the time and effort needed to train dual tasks [16,28,29]. However, recent evidence has shown the benefits of DTT in people with mild-to-moderate dementia [30,31]. Therefore, it is recommended to integrate DTT using a graded approach tailored to each person’s profile [28].

Ultimately, applying DTT to individuals with less favourable profiles depends upon the treatment strategy applied. Professionals and patients should make a joint decision on which treatment strategy to use (and how to progress), taking into account the capabilities of the patient, disease stage, age, and presence of other limitations. Commonly, professionals will combine both tasks only when single tasks are executed fluently and will make them more difficult gradually. Nevertheless, experiential expertise should be complemented by an educational component to be truly effective in delivering care to people with specific symptoms and profiles.

 

*Managing medication complex and fluctuating effects*

On and off fluctuations can also occur suddenly and unexpectedly, making them difficult to predict and manage during training sessions [3,32,33]. Alongside medications not providing sufficient motor improvement, there are also the side effects such as nausea and dyskinesia, as well as non-motor fluctuations (e.g., cognitive decline, anxiety, fatigue) related to OFF periods that reduce patients’ capacity to dual task. Again, clinical expertise that allows professionals to identify these fluctuations, abrupt changes in motor performance (with unpredictable offs, excessive movement dyskinesia), cognitive changes (with delirium, hallucinations, aggressiveness) and changes of consciousness and arousal (fainting, extreme sleepiness) will help therapists to adjust the interventions quickly and appropriately.

Additionally, although research recommends that people perform physiotherapy and exercise during their optimally medicated times [4], being able to coordinate treatment scheduling with best motor state can pose as a challenge in most settings. Organization’s schedules need to reflect sensitivity to ON & OFF phenomena and have the flexibility to book, rebook, and adjust treatment hours in an agile manner.

 

*Patient Motivation and Compliance*

Patients’ motivation with training was identified as a key difficulty experienced by a number of professionals. Motivation is expected to have direct impact on compliance with any treatment option; however, compliance is particularly difficult in people with PD [34,35,36].

Patients’ motivation and compliance with DDT is compromised due to commonly-reported factors that may interfere with patients’ ability to participate in any exercise, including the following: fluctuations in health from day to day, concerns about becoming injured or falling, insufficient expertise from professionals, time, and lack of support from care partners [37,38,39]. Strategies to bypass these barriers are critical to regain patients’ confidence in practice.

Patient education and engagement through improvement in their health literacy could be expected to enhance this compliance [40]. By sharing information regarding benefits of such trainings with patients and their caregivers/family, professionals can enable people with PD to manage their expectations and improve their compliance to DTT.

 

*Training Dynamics regarding tailoring Interventions and keeping it challenging and engaging*

Treatment strategies tailored to the individual achieves better clinical outcomes while increasing adherence and satisfaction [41]. Specific training, adequate ongoing educational support and regular, continuous experience applying DTT to people with PD is needed to practice in a manner supported by the emerging evidence [42]. Professionals should be able to personalize their interventions to each person’s needs, thus increasing the likelihood of keeping training sessions challenging and engaging for people with PD. For example, when choosing the DTT interventions professionals must consider: Which type of task most affects the patient’s complaint? Which one challenges the patient more? Which one facilitates the patient’s performance more? Which effect is the clinician attempting to invoke? Such reflections are key to place the person at the centre of care and enhance compliance to training programs [43]. Additionally, to be able to personalize DDT, healthcare and exercise professionals will need the knowledge and skills to adapt the training to each person.

Adjusting exercises per each person’s physical and cognitive abilities, can be a challenge, especially in a group setting. Consideration of the group’s overall severity of motor and cognitive impairments in conjunction with the complexity of dual task activities that will be required, in addition to overall environment challenges. Professionals may consider common physical problems in Parkinson (e.g., sit to stand, walking, stepping in place) and gradually add in complexity with a secondary task at a speed that everyone can follow. Modifying the gym environment in order to facilitate integrating dual task such as designated areas for groups close to walls that can be used for cueing to minimize problems with the reducing amplitude as well as minimizing the impacts of performing DTT in a noisy, distracting environment.

Participants also report struggling to continuously invent new exercises (or adapting older ones) in way that challenging and engaging for people with PD while continuing to be clinically meaningful. Given the current lack of evidence and protocols, exchanges with colleagues, including colleagues on social media platforms are a good source of new ideas.

 

*Maintaining patient safety when using DTT*

Safety issues during training are a critical concern. Importantly, when a person with PD is required to perform two tasks or divide attention between tasks, like in DTT, the lack of sufficient attentional resources results in the decrement in one or both concurrent tasks, with an increased risk of losing balance and falling [44,45,46]. Notably, there is an optimal level of disease-specific expertise necessary to integrate dual task practice, particularly with respect to being able to safely integrate exercises without putting patients at risk of falls and other possible adverse issues [4,47]. By carelessly or prematurely applying this type of exercise approach to people with PD, health or exercise professionals with a lack of professional expertise, may (a) increase risk of falls and other adverse events, (b) deliver unnecessary procedures, (c) include patients with less favorable profiles in these practices, (d) foster unrealistic expectations, and ultimately (e) compromise long term benefits and adherence [48]. Experience working with PD populations is a critical source of expertise in and of itself [42,49]; however, an effective educational component is necessary to complement this experiential expertise. It is critical to enhance expertise among professionals regarding knowledge of PD, interference effects of DTT, which types of benefit different exercises may provide to specific phenotypes of PD, and participant characteristics that influence people’s willingness to participate these exercise programs, as well as strategies to guarantee safety. Health-care professionals are accountable for their actions, therefore ensuring safe practices should be a priority [50,51].

Professionals can use several strategies that facilitate and reduce or avoid risks, such as using safety harness, keeping close to the patient, doing a slower progression on added tasks, giving clear instructions for the activity and using cueing strategies to maintain good performance. However, in a group setting this might be more challenging. Effective DTT in group formats is applicable depending on the amount of help available. According to guidelines [4], a maximum number of eight group participant per therapist is recommended. In situations where there is no additional help, starting with the most representative of most patients’ functional challenges in daily life (Walking/Transfer/ADL) and progressing slowly from simple to more complex tasks might be advisable. In certain contexts, working in the sitting position might be the only recourse to assure safety while adding in cognitive challenges.

 

*External Factors*

Several external factors also played a role in the challenges identified, namely insurance coverage, environmental issues and lack of family support.

Patients identified that insurers limited the number and time of training sessions for each patient. These limitations may reduce overall benefits, as people with PD need ongoing treatment to maintain the results and stay well [4,52]. Additionally, the amount of time available to personalize DTT to best suit the speed of progression for each individual may be critical. People with more severe disease, cognitive impairment or freezing of gait might need to progress slower and reduction in the overall quantity of clinical intervention may limit gains in a cohort that is already likely to have difficulty achieving and maintaining benefits from treatment [16,29]. In addition, healthcare and exercise professional must have a thorough knowledge of the individual to be able to personalize training effectively, and this can only come with spending time with patients [53].

Environments that facilitate DTT are also critical. Busy open space gyms can compromise teaching and learning of the patients. Finding quiet designated spaces for dual task away from walking passages can be difficult but will allow the patient to be more focused and not easily distracted by other people passing by.

The lack of family support was also identified as a difficulty that is critical to increasing the patient’s motivation. Studies have shown that social support network and emotional support from friends and family may predict adherence in the clinic and home setting [54]. Supporting families’ awareness of the evidence and benefits of such training are expected to play an important role in the successful care.

 

*Study strengths and limitations*

Rigorous web-based surveys are considered valuable for producing faster evidence than more traditional approaches [55]. However, they also pose some limitations. Selective participation could be one such limitation due to the automatic exclusion of potential participants who do not use social media platforms. This could arguably have added depth to the understanding of these challenges but does not necessarily bring on new dimensions, as participants’ responses were very consistent. In addition, we have to point out the low rate of non-American responses to the survey.

Another limitation of this study is that the open-ended question responses could not be explored with immediate follow-up questions that would allow deepening the knowledge on the subject. This motivated us to go deeper into qualitative data that would ultimately allow us to get a better understanding of these challenges, not yet explored. Researchers discussed every step made during the analysis process and discussions were held until consensus was reached when faced with different decisions. The decision-making process was detailed to allow others to follow the research. Descriptions with appropriate quotations were made available for readers who seek to incorporate this research’s findings to their site can judge transferability. External researchers examined methodically the finding to search for discrepancies, balancing their perceptions with the other researchers’ perceptions.

## 5. Conclusions

Our results revealed several difficulties experienced by healthcare and exercise professionals to integrate DTT in people with PD. According to participants’ reports, we identified ten categories of difficulties, namely PD signs and symptoms, medication, poor compliance, motivation, tailored interventions, patient safety, keeping it challenging and engaging, insurance coverage, environment and lack of family support.

Professionals remain challenged to integrate DTT into PD clinical care mainly due to difficulties in managing the chronicity of the disease and lack of patient adherence with exercises, in conjunction with external factors such as reimbursement and structure of the training environment.

## Figures and Tables

**Table 1:** Characteristics of the participants.

	Overall (N = 205)
Age (years)	
Mean (SD)	45.9 (11.1)
Median [Min, Max]	46.0 [23.0, 73.0]
Continent	
America	172 (83.9%)
Asia	2 (1.0%)
Australia	5 (2.4%)
Europe	26 (12.7%)
Discipline	
Athletic trainer and related	14 (6.8%)
Exercise physiology	6 (2.9%)
Fitness	2 (1.0%)
Medicine	3 (1.5%)
Neurology	2 (1.0%)
Occupational therapy	24 (11.7%)
Physiotherapy	141 (68.8%)
Speech language pathology	13 (6.3%)
Education	
Doctorate	73 (35.6%)
Masters	74 (36.1%)
Post Secondary/Undergraduate	58 (28.3%)
Years of Practice	
Mean (SD)	19.2 (11.1)
Median [Min, Max]	19.0 [1, 45.0]
Years of practice with Parkinson’s disease patients	
Mean (SD)	12.5 (8.8)
Median [Min, Max]	10.0 [1, 40.0]
Parkinson specific training	
No	17 (8.3%)
Yes	188 (91.7%)
Treatments setup	
Individual one on one	121 (59.0%)
Group setting	7 (3.4%)
Both	77 (37.6%)

**Table 2:** Difficulties faced by healthcare and exercise professionals when providing DTT with people with PD.

Theme	Category	Participants (N = 205)
Managing the disease and medication	PD signs and symptoms	104
	Medication	12
Patient dynamics	Poor compliance	68
	Motivation	27
Training dynamics	Tailored interventions	32
	Patient safety	16
	Keeping it challenging & engaging	10
External factors	Insurance coverage	10
	Environment	9
	Lack of family support	7

## Data Availability

The data presented in this study are available on request from the first author.
